# ROS/MMP-9 mediated CS degradation in BMSC inhibits citric acid metabolism participating in the dual regulation of bone remodelling

**DOI:** 10.1038/s41420-024-01835-5

**Published:** 2024-02-14

**Authors:** Wacili Da, Wen Jiang, Lin Tao

**Affiliations:** 1 Department of Orthopedics Surgery, Orthopedic Research Institute, West China Hospital, West China Medical School, Sichuan University, Chengdu, Sichuan Province China; 2 Department of Orthopedics, First Hospital of China Medical University, Shenyang, Liaoning China

**Keywords:** Osteoporosis, Glycobiology

## Abstract

It is necessary to figure out the abnormal energy metabolites at the cellular level of postmenopausal osteoporosis (PMOP) bone microenvironment. In this study, we constructed PMOP model by ovariectomy and identified 9 differential metabolites compared with control femur by energy metabolomic. The enrichment analysis of differential metabolites revealed that tricarboxylic acid cycle, glucagon pathway and purinergic signaling pathway were the main abnormal metabolic processes. Citric acid was identified as the key metabolite by constructing compound reaction–enzyme–gene network. The functional annotation of citric acid targets identified by network pharmacological tools indicated that matrix metalloproteinase 9 (MMP-9) may be involved in regulating citric acid metabolism in the osteogenic differentiation of bone marrow mesenchymal stem cell (BMSC). Molecular docking shows that the interaction forces between MMP-9 and citric acid synthase (CS) is −638, and there are multiple groups of residues used to form hydrogen bonds. Exogenous H_2_O_2_ promotes the expression of MMP-9 in BMSC to further degrade CS resulting in a decrease in mitochondrial citric acid synthesis, which leads to the disorder of bone remodeling by two underlying mechanisms ((1) the decreased histone acetylation inhibits the osteogenic differentiation potential of BMSC; (2) the decreased bone mineralization by citric acid deposition). MMP-9-specific inhibitor (MMP-9-IN-1) could significantly improve the amount of CS in BMSC to promote cellular citric acid synthesis, and further enhance bone remodeling. These findings suggest inhibiting the degradation of CS by MMP-9 to promote the net production of citric acid in osteogenic differentiation of BMSC may be a new direction of PMOP research.

## Introduction

Osteoporosis (OP) is a chronic disease characterized by systemic endocrine and metabolic disorders. Whether it is primary (caused by ageing or lack of sex hormones) or secondary (caused as a result of hyperthyroidism, diabetes, obesity, Cushing’s syndrome, anorexia, rheumatoid arthritis, drug effects, etc.), the root mechanism of its occurrence is the imbalance of bone remodelling homeostasis [1–3]. Among them, postmenopausal osteoporosis (PMOP) caused by estrogen deficiency is the most common type of osteoporosis [4]. With the ageing of the population, more than 50% of women over the age of 50 in the world have a significant risk of fracture [4]. Osteoporosis and fractures related to osteoporosis have become major public health problems and economic problems in the human population and have significantly increased the consumption of healthcare resources. Therefore, it is of great practical significance to further study the pathogenesis of postmenopausal osteoporosis and explore new intervenable targets.

The homeostasis of physiological bone remodelling is in dynamic balance and is susceptible to the external environment, including energy metabolism substrates, hormones and growth factors. Both osteogenesis of osteoblasts and bone resorption of osteoclasts depend heavily on energy consumption. In recent years, a number of studies on tumours, cardiovascular diseases and nervous system diseases have tried to explore their pathogenesis and treatment from the perspective of energy metabolism [5–8]. The root cause of osteoporosis is a disorder of the dynamic balance between bone formation and bone resorption. Moreover, the metabolism of various cells in bone, including glucose and lipid metabolism, fatty acid distribution, and amino acid content, is closely related to bone formation and bone resorption [9–12]. Focusing on the bone microenvironment, the energy metabolism disorder of osteoblasts and osteoclasts is the key factor of the disease. Therefore, we assume that some energy metabolites will have specific changes in the state of osteoporosis, and we can infer the pathological changes in related metabolic enzymes.

Therefore, energy metabolomics was applied in this study to identify specific differentially abundant metabolites of PMOP. At the same time, network pharmacology was used to predict the targets of differentially abundant metabolites and further analyse the molecules involved in the occurrence and development of osteoporosis. Based on the combined analysis of energy metabolomics and target prediction, we identified citric acid as the key metabolite. Interestingly, we predict that matrix metalloproteinase (MMP-9) is a potential control target for citric acid metabolism. It is well known that MMP-9 is involved in the regulation of bone remodelling, and at present, most attention has been given to the mechanism of collagen degradation by MMP-9 secreted by osteoclasts to promote bone resorption, and there is a lack of in-depth exploration on the regulation of MMP-9 in bone regeneration [13]. In combination with our previous research on the effects of citric acid on bone remodelling [14], citrate synthase (CS) is a key enzyme involved in the regulation of mitochondrial citric acid synthesis in BMSCs. In addition, it has been reported that CS is the degradation substrate of MMP-9 [15, 16]. Therefore, this study confirmed a new mechanism of postmenopausal osteoporosis in which oxidative stress in the bone microenvironment after menopause promotes the expression of MMP-9 in BMSCs and degrades CS, leading to a decrease in mitochondrial citric acid synthesis. On the one hand, it reduces the osteogenic differentiation potential of BMSCs by reducing histone acetylation. On the other hand, it will directly reduce the bone matrix deposition of citric acid, and the two factors together lead to the occurrence and development of postmenopausal osteoporosis.

## Results

### Evaluation of the mouse model and data results

**Fig. 1:**
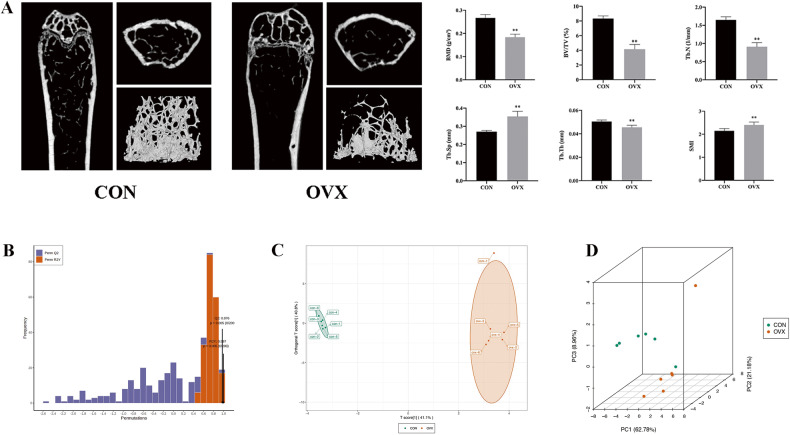
Data evaluation for energy metabolomics. **A** Micro-CT results of femur in different groups of mice, **B** OPLS-DA model validation, the model is the best when *p* < 0.05, **C** OPLS-DA model score plot of OVX group and CON group, **D** PCA analysis of metabolites, PC1 = 62.78%, PC2 = 21.18%, PC3 = 8.96%. **P < 0.01, Compared with control, CON control, OVX ovariectomized. BMD bone mineral density, BV/TV bone volume/tissue volume, Tb.N trabecular number, Tb.Sp trabecular separation, Tb.Th trabecular thickness, SMI structure model index.

As shown in Fig. 1A, ovariectomy can induce bone loss in mice and lead to bone loss. The BMD, BV/TV, Tb.N and Tb.Th of mice in the OVX group were significantly lower than those in the CON group, and the Tb.Sp in the OVX group was significantly higher than that in the CON group, accompanied by increased rod-shaped trabecular bone. In addition, we used the model established by the OPLSR Anal function to conduct 200 random permutation and combination experiments on the omics data to verify the stability and reliability of the model. The OPLS-DA model could well separate the expression levels of metabolites in the two groups of samples, and the *P* values of Q2 and R2Y were less than 0.05, indicating that the model was qualified (Fig. 1B, C). Moreover, PCA results (Fig. 1D) also showed that there was a significant difference in the overall expression of metabolites between the two groups.

### Identification and enrichment analysis of differentially abundant metabolites

**Fig. 2:**
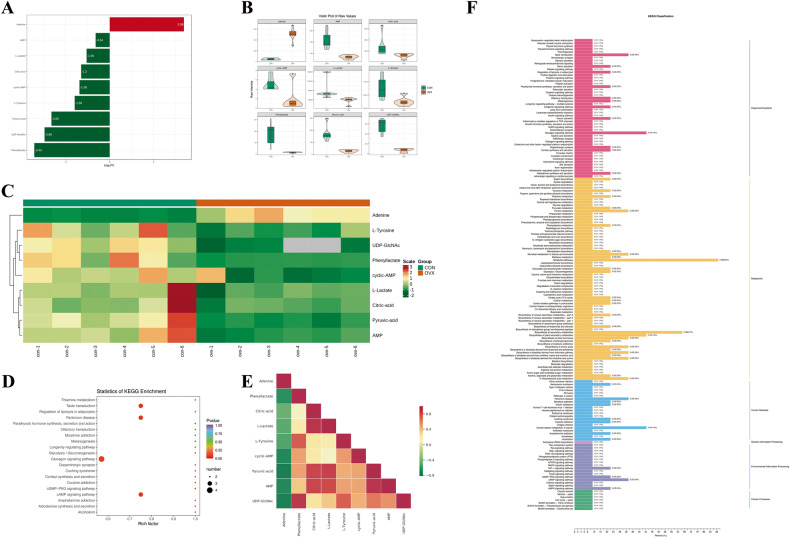
Differential metabolites identification and functional enrichment. **A** Fold changes of differential metabolites between OVX group and CON group, **B** Violin plot visualization of differential metabolites, the box in the middle represents the interquartile range, the black horizontal line in the middle is the median, and the outer structure represents the distribution density, **C** Cluster analysis of differential metabolites, **D** Significance analysis of KEGG annotation, the size of the circle represents the number of enriched metabolites, the colour of the circle represents the significance of the enriched pathway, **E** Correlation analysis between differential metabolites, red represents a positive correlation, green represents a negative correlation, **F** Classification of KEGG functional annotation, CON control, OVX ovariectomized.

A total of 9 metabolites in postmenopausal osteoporotic bone tissue showed significant changes compared with normal bone (Table 1), among which 8 were downregulated and 1 was upregulated. The levels of variation in differentially abundant metabolites were visualized (Fig. 2A, B). To facilitate the observation of metabolite changes, we normalized the differentially abundant metabolites and performed cluster analysis (Fig. 2C).

**Table 1:** Differential metabolites in postmenopausal osteoporosis compared with control.

Compounds	Class	VIP	P-value	Log2FC	Type
Adenine	Nucleotide metabolomics	1.515667	0.000261	3.3792	up
Phenyllactate	Organic Acid and Its Derivatives	1.379055	0.012614	−3.4313	down
Citric acid	Amino Acid metabolomics	1.031178	0.079983	−1.2997	down
L-Lactate	Organic Acid and Its Derivatives	1.002846	0.056083	−1.0511	down
L-Tyrosine	Amino Acid metabolomics	1.070575	0.03329	−1.592	down
cyclic-AMP	Nucleotide metabolomics	1.132321	0.035249	−1.3752	down
Pyruvic acid	Amino Acid metabolomics	1.455704	0.002543	−2.6511	down
AMP	Nucleotide metabolomics	1.352324	0.005884	−0.6398	down
UDP-GlcNAc	Nucleotide metabolomics	1.139238	0.000327	−2.9647	down

These differentially abundant metabolites mainly participate in 70 metabolic links, including purine metabolism, secondary metabolite synthesis, citric acid cycle, amino acid synthesis and carbon metabolism (Fig. 2F). In systemic organic systems, these differentially abundant metabolites were involved in a total of 48 items, and the glucagon signal system was the most significant (Fig. 2F, D). In addition, human disease functional annotation of differentially abundant metabolites showed that the most closely related metabolites were central carbon metabolism in cancer and Parkinson’s disease (Fig. 2F). Differentially abundant metabolites were mainly involved in the cAMP, AMPK, cGMP-PKG and HIF-1 signalling pathways (Fig. 2F). Moreover, we performed correlation analysis among the metabolites (Fig. 2E), in which adenine had a significant negative correlation with phenyllactate, cyclic AMP, pyruvate and AMP. However, phenyllactate and UDP-GlcNAc, citric acid and L-lactate, and pyruvate and AMP were positively correlated.

### Target identification of differentially abundant metabolites

**Fig. 3:**
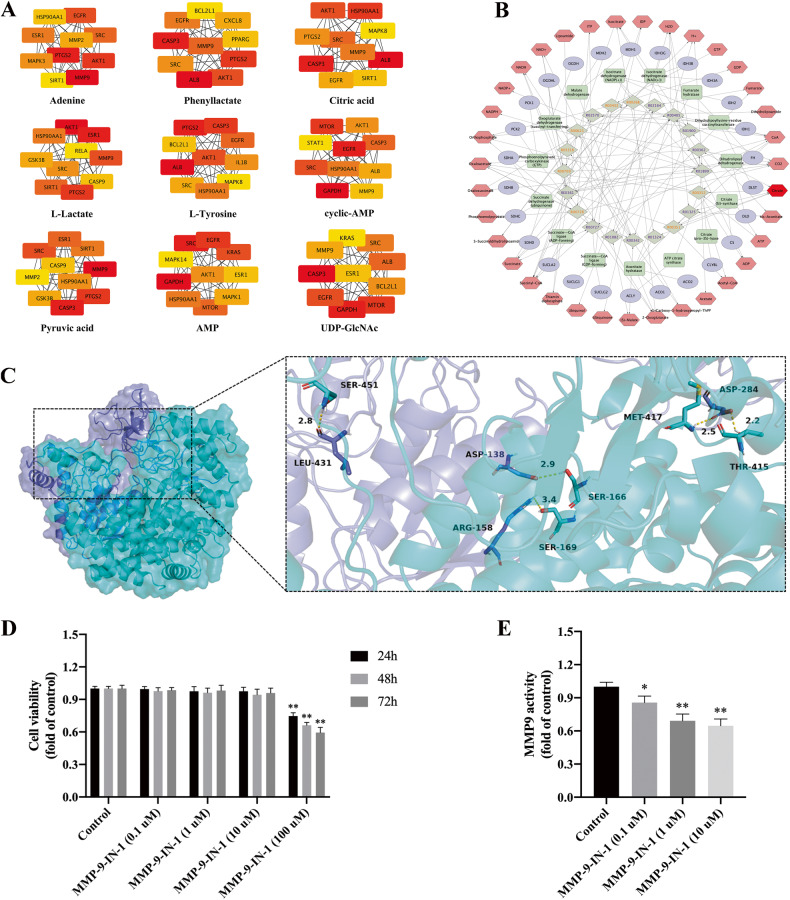
Topological network construction of targets of differential metabolites and key metabolite identification. **A** The top 10 hubgenes of each metabolite, **B** Compound-reaction-enzyme-gene network, hexagon is metabolite, ellipse is gene, rectangle is enzyme, and rhombus is metabolic reaction, the deeper colour indicates the higher importance of this substance in the network, **C** Molecular docking of MMP-9 and CS, **D** Effect of MMP-9-IN 1 on osteoblast proliferation, **E** MMP-9 activity. *P < 0.05, **P < 0.01, Compared with control.

Target prediction based on molecular structure was performed for differentially abundant metabolites. Due to the different screening logic and criteria, the number of predicted targets in each database is different. Therefore, we summarized each database to remove duplicate targets (Table 2). In addition, a total of 1294 pathogenic targets of PMOP (see the supplementary materials) were identified from five databases for intersection analysis with metabolite targets, among which L-tyrosine had the highest target repetition and L-lactate had the lowest. Figure 3A shows the topological analysis of the intersection targets of various metabolites, and it can be found that there are key genes, including PTGS2, MMP-9, HSP90AA1, ESR1, SRC, etc. Citric acid was identified as the key differentially abundant metabolite based on the compound-reaction-enzyme-gene network (Fig. 3B). Citric acid, as a key intermediate in the mitochondrial tricarboxylic acid cycle, is produced from acetyl-CoA and oxaloacetate by CS and converted to isocitrate under the catalysis of mitochondrial aconitase (M-acon). Then, it is oxidized to α-ketoglutarate by isocitrate dehydrogenase 2 and finally achieves oxidative phosphorylation for energy supply. Therefore, from the perspective of citric acid synthesis, we have two directions to further study the mechanism of promoting the net accumulation of citric acid, namely, the promotion of CS and the inhibition of M-acon.

**Table 2:** Target prediction of differential metabolites.

Compounds	SEA	STITCH	TargetNet	SwissTargetPrediction	PharmMapper	Total
Adenine	9	10	277	43	104	347
Phenyllactate	283	1	263	100	248	604
Citric acid	22	8	253	64	292	523
L-Lactate	3	0	273	10	84	299
L-Tyrosine	286	6	253	100	294	638
cyclic-AMP	176	0	80	100	255	442
Pyruvic acid	3	74	306	8	128	366
AMP	246	15	59	100	295	506
UDP-GlcNAc	146	1	115	75	293	472

### MMP-9 is a crucial target of citric acid metabolism

It has been reported that approximately 90% of the citric acid in the human body exists in bone, which is specifically produced by osteoblasts differentiated from BMSCs, and osteoblasts integrate citric acid into bone in the form of mineralization [17–20]. Therefore, we annotated the top 10 targets and collected the drugs targeting these hub genes (Table 3). We found that the SIRT1 and MMP-9 genes were closely associated with citric acid. Notably, CS has been reported to be a substrate of MMP-9 [15, 16]. By means of protein‒protein interaction analysis in PyMOL (Fig. 3C), all functional residues were identified and classified according to their interactions. In the hydrogen bonding interaction, there are multiple groups of residues used to form hydrogen bonds between MMP-9 and CS, such as the hydrogen bond formed by Asp138 of MMP-9 and Ser166 of CS. With these interaction forces, the scoring of MMP-9 and CS is -638, which is a good performance. Moreover, MMP-9 is also involved in the degradation of bone collagen in PMOP. Therefore, we infer that MMP-9 degrades CS in BMSCs, resulting in reduced citric acid secretion and bone mineralization. Effectively promoting citric acid synthesis and secretion in BMSCs and osteoblasts derived from BMSCs by inhibiting MMP-9 and correcting bone remodelling homeostasis will contribute to the treatment of postmenopausal bone loss. We identified a specific inhibitor of MMP-9 (MMP-9-IN 1) (Table 3) and found that MMP-9-IN 1 (≤10 µM) had no significant effect on the proliferation of BMSCs (Fig. 3D), but it significantly inhibited MMP-9 activity (Fig. 3E).

**Table 3:** The hub genes of citric acid and targeted drugs.

Rank	Target	Full name	Drug
1	ALB	albumin	Patent Blue
2	CASP3	caspase 3	AZ-10417808, NQDI-1, PAC-1, PETCM, glycyrrhizic acid
3	HSP90AA1	heat shock protein 90 alpha family class A member 1	CCT018159, EC-144, VER-49009, alvespimycin, gedunin, geldanamycin,
4	AKT1	AKT serine/threonine kinase 1	A-674563, API-1, SB-747651A
5	SRC	SRC proto-oncogene, non-receptor tyrosine kinase	AZM-475271, KB-SRC-4, PD-166285, PP-2, TG-100572, WH-4-023, dasatinib, tirbanibulin, bosutinib, dasatinib, 1-naphthyl-PP1, 3,4-methylenedioxy-beta-nitrostyrene
6	MMP9	matrix metallopeptidase 9	CP-471474, UK-356618, CTS-1027, MMP-9-IN-1, MMP2-I1, salvianolic acid B, abametapir, cipemastat, ilomastat, marimastat
7	PTGS2	prostaglandin-endoperoxide synthase 2	SC-236, carprofen, deracoxib, etodolac, etoricoxib, firocoxib, propacetamol, tiaprofenic acid, vedaprofen, asaraldehyde, rutaecarpine
8	EGFR	epidermal growth factor receptor	AZ5104, BIBU-1361, CGP-52411, CNX-2006, OSI-420, PD-158780, WZ-3146, WZ8040, tyrphostin-AG-494, cetuximab, panitumumab, gefitinib, icotinib, osimertinib
9	SIRT1	sirtuin 1	SRT1720, sirtinol, splitomycin, tenovin-6, cambinol
10	MAPK8	mitogen-activated protein kinase 8	BI-78D3, SR-3306, SU-3327, pyrazolanthrone

### MMP-9-IN 1 promotes citric acid metabolism in BMSCs and bone formation in vivo

**Fig. 4:**
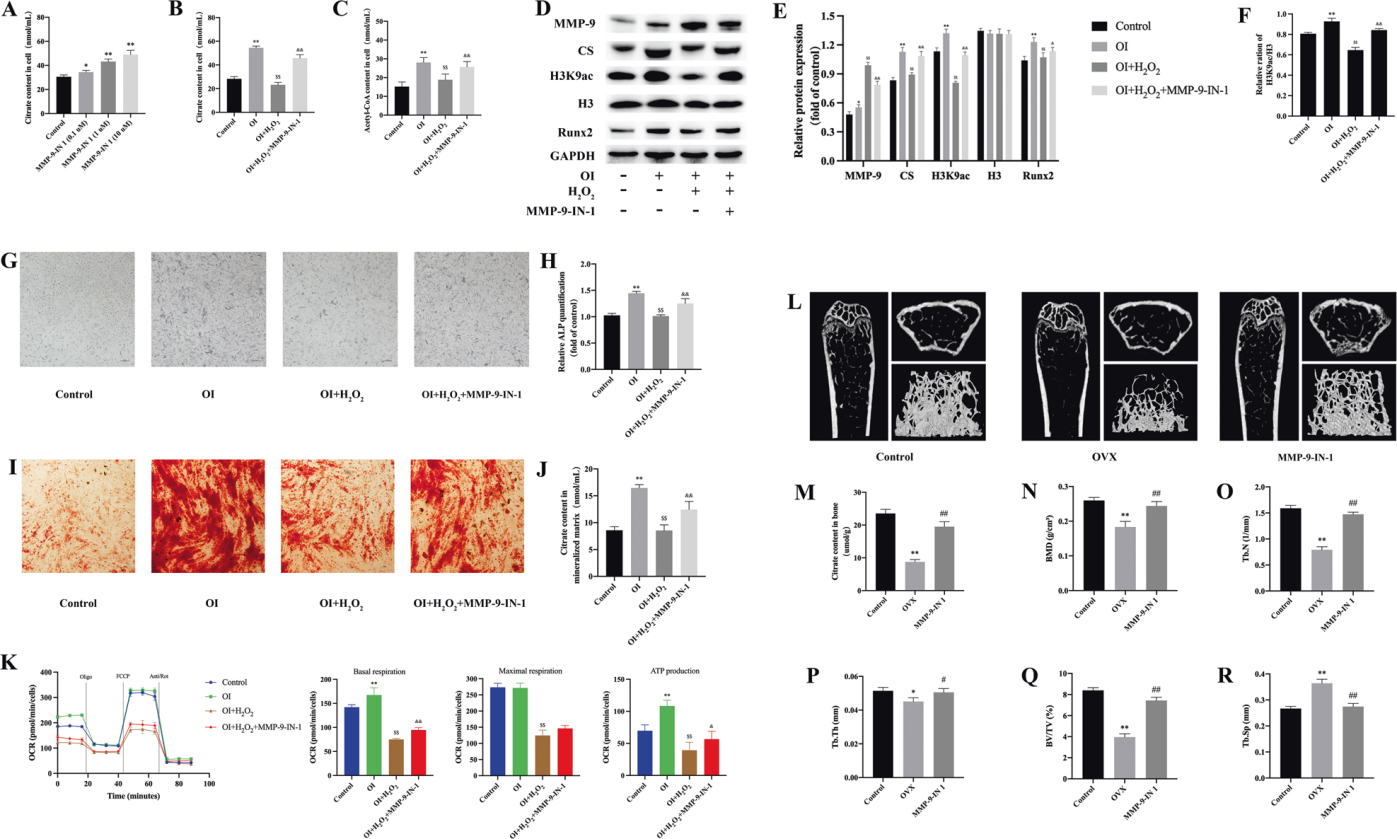
MMP-9-IN 1 promotes citric acid secretion in BMSCs and bone formation in vivo. **A**–**C** Effect of MMP-9-IN 1 and H_2_O_2_ on citric acid and acetyl-coA, **D** Western blot of MMP-9(78 Kd), CS(45 Kd), H3K9ac(17 Kd), H3(17 Kd), and Runx2(60 Kd), **E** Relative expression of MMP-9, CS, H3K9ac, H3 and Runx2, **F** Relative expression of H3K9ac/H3, **G**, **H** ALP staining and quantification, **I,** **J** Alizarin red staining and citric acid quantification, **K** oxygen consumption rate of BMSCs, **L** Micro-CT results of femur, **M** Citric acid per unit bone, **N**, **R** Parameter analysis of Micro-CT. *P < 0.05, **P < 0.01, compared with control; $P < 0.05, $$P < 0.01, compared with OI; & *P* < 0.05, & *P* < 0.01, compared with OI + H_2_O_2_, #*P* < 0.05, ##*P* < 0.01, compared with OVX; H3K9ac, acetylated histone H3K9.

Decreased citrate synthesis in mitochondria of osteoblasts differentiated from BMSCs may result in reduced citrate incorporation into the mineralized matrix. In addition, mitochondrial citric acid can be converted into acetyl-CoA by ATP citrate lyase after being exported to the cytoplasm through CTP. The amount of acetyl-CoA in the cytoplasm is positively correlated with the level of histone acetylation [21, 22]. It has been reported that the level of histone acetylation upregulated in BMSCs can enhance the expression of runt-related transcription Factor 2 (Runx2) and bone morphogenetic proteins (BMPs) and ultimately promote the osteogenic differentiation of BMSCs [23, 24]. As shown in Fig. 4A, MMP-9-IN 1 significantly increased the citric acid content in BMSCs. Exogenous H_2_O_2_ interference was used to simulate the oxidative stress of the bone microenvironment after menopause, resulting in a significant increase in the expression of MMP-9 and a decrease in CS, osteogenic differentiation protein expression, histone acetylation (Fig. 4D–F), and citric acid and acetyl-CoA secretion in BMSCs, which can be significantly reversed by MMP-9-IN 1 (Fig. 4B, C). Moreover, MMP-9-IN 1 restored osteogenic matrix formation in osteoblasts, including citrate deposition, by inhibiting the activity of MMP-9 (Fig. 4G–J). To investigate mitochondrial function in response to the addition of H_2_O_2_ and MMP-9-IN 1, BMSCs were subjected to measurements of mitochondrial oxidative capacities through oxygen consumption rate (OCR) evaluation (Fig. 4K). The MMP-9-IN 1 condition significantly increased basal respiration (OI + H_2_O_2_: 75.46 ± 6.28 OCR, OI + H_2_O_2_ + MMP-9-IN-1: 94.6 ± 7.2 OCR) and mitochondria-linked ATP production (OI + H_2_O_2_: 39.33 ± 13.61 OCR, OI + H_2_O_2_ + MMP-9-IN-1: 52.64 ± 15.32 OCR) compared to H_2_O_2_ stimulation. Altogether, these results confirmed that MMP-9-IN1 can significantly improve the efficiency of the tricarboxylic acid (TCA) cycle in BMSCs induced by oxidative stress injury and further enhance mitochondrial function. In vivo studies confirmed that MMP-9-IN 1 could improve bone health, including BMD, BV/TV, Tb.N and Tb.Th, in ovariectomized mice and reduce trabecular bone separation and MSI (Fig. 4L–R). Moreover, it is worth noting that MMP-9-IN 1 can also restore citric acid in bone (Fig. 4M), which is consistent with the results of bone mass and in vitro experiments.

## Discussion

Disturbances in basic physiological and biochemical processes, such as lipid metabolism, energy metabolism, amino acid metabolism and bile acid metabolism, are important factors that affect the biological efficiency of cells. Bone tissue is mainly composed of inorganic minerals, organic collagen fibres, osteoblasts, osteoclasts and osteocytes. The composition and content of metabolic substrates in the bone microenvironment regulate the metabolic state and phenotypic changes in various cells, such as differentiation, proliferation, apoptosis, and autophagy. It is well known that osteoblasts differentiated from BMSCs are responsible for the generation of new bone in bone remodelling homeostasis, while osteoclasts derived from monocyte macrophages are responsible for the absorption of old bone, and these two biological processes are highly dependent on ATP consumption [25–27]. Bone remodelling homeostasis is essential for maintaining bone mass, calcium balance and bone strength. Therefore, our energy metabolomics of osteoporotic femurs revealed abnormal metabolites in the pathological state.

Glycolysis, the TCA cycle and the pentose phosphate pathway were the main metabolic processes in which differentially abundant metabolites were enriched. We know that cAMP produced by adenylate cyclase catalysing ATP can act as an intracellular second messenger, which can participate in signal transduction and regulate gene expression. The decreased cAMP may be due to abnormal adenylate cyclase activity or low ATP in bone. Oxidative phosphorylation and glycolysis are two important metabolic pathways needed for ATP production to maintain bone remodelling homeostasis [7]. Although our study only identified differences in L-tyrosine, it also suggested that abnormal amino acid content is closely related to the occurrence of osteoporosis. Some studies have found that the serum L-tyrosine of ovariectomized rats is higher than that of the control group [28], which is contrary to our research results, indicating that different tissue localization of L-tyrosine may play different roles. Aromatic amino acids (tyrosine, tryptophan, arginine, and phenylalanine) can activate different anabolic signalling pathways in BMSCs [29, 30]. Refaey et al. proposed that abnormal oxidation of tyrosine could inhibit the proliferation and osteogenic differentiation of BMSCs, which may play a pathogenic role in ageing-induced bone loss [31]. In addition to being a direct component of protein synthesis, amino acids are important energy substrates and essential carbon and nitrogen donors. There is evidence that glutamine is decomposed to form α-ketoglutarate, which enters the TCA cycle and is converted to citric acid [32, 33]. In short, amino acids may be directly involved in energy metabolism pathways and may also be involved in amino acid-dependent transcription and synthesis of related cofactors in the TCA cycle. It is noteworthy that osteoporotic bone tissue is rich in adenine. Purines and pyrimidines are ubiquitous in animal cells and are not only components of nucleic acids but also involved in intracellular biochemical reactions and energy transmission [34]. Purinergic signals regulate many biological behaviours of cells, including proliferation and differentiation, chemotaxis, cytokine release, reactive oxygen species production, cell fusion, phagocytosis, apoptosis, necrosis, and inflammation [35–37]. At present, due to the diversity of purine receptor subtypes, the specific effects of purine metabolism on osteoblasts and osteoclasts are still under exploration.

Surprisingly, citric acid was the key substance among the nine different metabolites, although it was not the most variable. Aerobic glycolysis in osteoblasts may be combined with the active secretion of citric acid, which is essential for the formation of nanohydroxyapatite crystals [17]. Citric acid derived from osteoblasts participates in bone matrix mineralization and promotes bone regeneration [18–20]. Citric acid efflux from osteoblasts may imply elevated mitochondrial citric acid, thereby inhibiting pyruvate entry into the TCA cycle. Studies have shown that thiazolidinediones can bind to and inhibit pyruvate transporters. Although thiazolidinediones such as rosiglitazone are used as antidiabetic drugs, these drugs also cause bone loss [38, 39]. It can be considered that in the process of normal bone remodelling, osteoblasts actively transfer the net citric acid in mitochondria to the cytoplasm, which not only ensures the inorganic ions involved in bone mineralization but also ensures that pyruvate smoothly enters the TCA cycle to provide ATP.

**Fig. 5:**
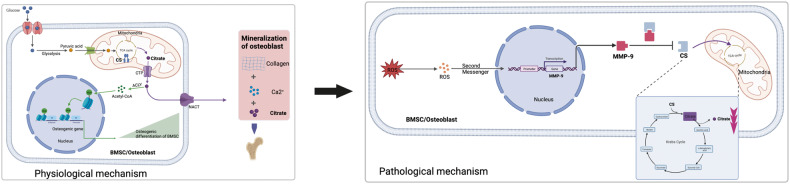
Citric acid metabolism-mediated osteogenic differentiation and mineralization of BMSCs. Postmenopausal oxidative stress microenvironment promotes the expression of MMP-9 in BMSCs to degrade CS, resulting in a decrease in net citric acid synthesis. On the one hand, the reduced citric acid impairs the indirect acetyl-CoA output of cytoplasm, resulting in reduced histone acetylation and reduced osteogenic differentiation potential of BMSCs. On the other hand, citric acid is directly involved in reduced binding to collagen and Ca^2+^ in the extracellular matrix, resulting in reduced bone mineralization. Acetyl-CoA acetyl coenzyme A, ACLY ATP-citrate lyase, CS citrate synthase, CTP mitochondrial citrate transporter, MMP-9 matrix metalloproteinase 9, NACT, Na+/citrate cotransporter, ROS reactive oxygen species.

Focusing on citric acid metabolism, the bone deposition of citric acid depends on the net production of osteoblasts, and this process mainly depends on the following molecules: CS participates in the synthesis of citric acid, and M-acon promotes the conversion of citric acid to isocitric acid, which is inhibited by zinc and the mitochondrial citrate transporter (CTP) [40–43]. Citric acid production increased with osteoblast differentiation in vitro, accompanied by changes in the expression of proteins related to citrate secretion (upregulated: CS, CTP, downregulated: M-acon), suggesting that osteoblasts are specific cells in bone that produce citric acid. It is worth noting that nonosteogenic BMSCs do not secrete citric acid [44]. Our previous study found that the decreased expression of ZIP-1 in osteoporotic osteoblasts leads to the reduced transport of zinc to mitochondria, which leads to the enhanced activity of M-acon and the decreased net secretion of citric acid [14]. In this study, we were surprised to find MMP-9, a potential target of citric acid metabolism. Studies have reported that osteoporotic bone (compared with normal bone) tissues exhibit higher levels of MMP-9 [45]. MMP-9 in the osteoporosis microenvironment is mainly involved in the degradation of collagen and proteoglycans [46]. Notably, CS is also a potential substrate for MMP-9 degradation [15, 16]. Our study also found that an MMP-9-specific inhibitor (MMP-9-IN-1) could significantly rescue the amount of CS in BMSCs, promoting cellular citric acid synthesis and restoring bone mass. We believe that the oxidative stress microenvironment activates the expression of MMP-9 in postmenopausal osteoporotic BMSCs to further degrade mitochondrial CS, resulting in a reduction in mitochondrial citric acid synthesis. There are two potential causative mechanisms for bone remodelling disorder mediated by reduced citric acid (Fig. 5): (1) Indirectly reduced acetylation of cytoplasmic histone inhibits the osteogenic differentiation potential of BMSCs; alternatively, (2) Decreased net citric acid secretion in osteoblasts derived from BMSCs reduces bone mineralization directly.

## Conclusion

Overall, the dysregulation of energy metabolites in the bone microenvironment is responsible for the abnormal function and phenotype of cells, which further leads to an imbalance in bone remodelling. The TCA cycle, glucagon pathway and purinergic signalling pathway are the key loops of metabolic disorders. There is much room to explore the specific effects of specific molecules on specific cells, and that is where our future research will focus. Finally, reducing the degradation of CS by MMP-9 to promote the production of net citric acid in the osteogenic differentiation of BMSCs may be a new direction of bone research.

## Materials and Methods

### Animals and cells

All animal experiments were approved by the Animal Care and Use Committee of the First Affiliated Hospital of China Medical University. Ten-week-old female SPF grade C57BL6/J mice were purchased from the animal research centre of the Chinese Academy of Sciences. Mice were randomly divided into 4 groups (6 mice in each group): control group (CON), sham operation group (Sham), ovariectomy group (OVX) and ovariectomy+MMP-9 inhibitor group (OVX + MMP-9-IN 1 (MedChemExpress, New Jersey, USA, PubChem CID: 135415473)). Mice in the sham-operated group underwent dorsal skin excision and periovarian fat excision, while mice in the OVX group underwent bilateral oophorectomy after anaesthesia with an animal anaesthesia machine (oxygen flow was 0.5 L/min, isoflurane inhalation concentration was 1%-3%). The OVX + MMP-9-IN 1 group was intraperitoneally injected with MMP-9-IN 1 three days after ovariectomy (2 mg/kg [47], twice a week, for 8 weeks), and the other mice were injected with the same dose of phosphate-buffered saline (PBS). WD was responsible for the group design of animal experiments, and WJ and LT were responsible for the data analysis. Primary BMSCs were obtained from the mouse femur under sterile conditions. BMSCs were recently authenticated by western blot and flow cytometry, and tested for mycoplasma contamination. Conventional osteogenic medium (10 nM dexamethasone, 50 mg/ml ascorbic acid and 10 mM β-glycerophosphate (all from Sigma, St. Louis, USA)) was used to induce BMSCs to differentiate into osteoblasts. Cells were specifically divided into BMSC; BMSC + MMP-9-IN 1 (0.1-1-10-100 µM) and BMSC; BMSC+ osteogenic induction (OI); BMSC + OI + H_2_O_2_; BMSC + OI + H_2_O_2_ + MMP-9-IN 1 (10 µM). In addition, H_2_O_2_ (200 µM) was used for oxidative stress stimulation for 30 min. The medium was changed every two days.

### Metabolomics experiment

After 8 weeks of feeding, the mice were sacrificed by cervical dislocation, and the limbs were collected. The femur and tibia were washed with PBS and homogenized. After that, 50 mg of homogenized sample was accurately weighed into a new EP tube, and 500 µL of 70% methanol/water extract (precooled at −20 °C) was added. After gradient centrifugation, 200 µL of supernatant was removed for analysis. Metabolite determination was carried out using ultrahigh-performance liquid chromatography–tandem mass spectrometry (LC‒MS/MS, QTRAP 6500+, SCIEX, USA).

### Data processing and difference analysis

Data analysis mainly depends on R software (https://www.r-project.org/), including principal component analysis (PCA), violin chart visualization, orthogonal partial least squares discriminant analysis (OPLS-DA) and Pearson correlation coefficient analysis. After log2 conversion, the original data were processed by mean centering and then analysed with the help of the OPLSR Anal function of the MetaboAnalyst R package. The differentially abundant metabolites were screened by combining the fold change, p value and variable importance (VIP) value of OPLS-DA. Screening criteria: fold change ≥ 1.5 and fold change ≤ 0.66 or p value < 0.05 in the two-tailed Student’s *t*-tests or VIP ≥ 1.

### Enrichment analysis and target identification of differentially abundant metabolites

The online database KEGG (http://www.genome.jp/kegg/) was used for functional annotation and enrichment analysis of differentially abundant metabolites. The structural information (3D structure format (SDF) and canonical SMILES sequence) of the differentially abundant metabolites was obtained from the PubChem database (https://pubchem.ncbi.nlm.nih.gov/). Structure-based target prediction is performed with the help of network pharmacological tools, including PharmMapper, SEA, STITCH, SwissTargetPrediction and TargetNet. Second, the DisGeNET, DrugBank, GeneCards, OMIM and TTD databases were used to obtain the disease targets of osteoporosis with the retrieval strategy of “postmenopausal osteoporosis”. The intersection analysis of targets between each metabolite and PMOP was performed. The interaction network analysis of the intersection by each metabolite was constructed on STRING. Then, the cytoHubba plug-in in Cytoscape was used to obtain the top 10 hub genes of each metabolite. Then, we constructed a compound-reaction-enzyme-gene network by MetScape to identify key differentially abundant metabolites, metabolic pathways and enzymes. In addition, we collected drugs that can target the top 10 hubgenes through the REPURPOSING module of the Connectivity Map and DrugBank databases.

### Molecular docking

The X-ray crystal structures of MMP-9 and CS were retrieved from the Protein Data Bank. To ensure the accuracy of the docking results, the protein was prepared by AutoDockTools-1.5.7, the water molecules were manually eliminated from the protein, and polar hydrogen was added. The Docking Web Server (GRAMM) was used for protein-protein docking. The resulting protein-protein complex was also manually optimized by removing water and adding polar hydrogen by AutoDockTools-1.5.7. Finally, the protein-protein interactions were predicted, and the protein-protein interaction figure was generated by PyMOL. The MMP-9 protein is represented as a slate cartoon model, the CS protein is shown as a cyan cartoon model, and their binding sites are shown as pink stick structures. When focusing on the binding region, the binding site is then shown as a presentation of the protein to which it belongs.

### Cell viability assay

Cells were seeded in 96-well plates (Thermo Fisher Scientific, MA, USA) at 1 × 10^4^ cells/well for 24 h and then treated with MMP-9-IN 1 (0, 0.1, 1, 10 µM) for 24, 48 and 72 h. Cell viability was determined using a cell counting kit-8 (CCK-8) assay (Dojindo Molecular Technologies, Inc.). The absorbance was measured at 450 nm using an ELISA microplate reader (Bio‑Rad).

### MMP-9 activity detection

Cells were seeded in 96-well plates at 1 × 10^4^ cells/well for 24 h and then treated with MMP-9-IN 1 (0, 0.1, 1, 10 µM) for 72 h. The activity of MMP-9 was determined at 420 nm in a fluorescence microplate reader according to the procedure of the MMP-9 inhibitor screening detection kit (Abcam, Shanghai, China).

### Western blot

Cells were seeded in 6-well plates at 1 × 10^6^ cells/well for 24 h and then treated with MMP-9-IN 1 (0, 0.1, 1, 10 µM) for 1 week. Then, 20 µl of RIPA lysate was added to each plate, after which the cell lysate was scraped and collected into 1.5 ml EP tubes. Cells were completely lysed by sonication, and after centrifugation at 14,000 r/min for 30 min at 4 °C, the supernatant was transferred to a new 1.5 ml EP tube for BCA (Generay, Shanghai, China) protein quantification. Gel electrophoresis was performed with a protein loading of 15 µg. The primary antibody was incubated at 4 °C overnight (CS (#14309, 1:1000, CST, USA); MMP-9 (ab283575, 1:1000, Abcam, USA); Histone H3 (#4499, 1:2000, CST, USA); Acetyl-H3K9 (#9649, 1:1000, CST, USA); Runx2 (#12556, 1:1000, CST, USA) and GAPDH (ab181602, 1∶10000, Abcam, USA)), and the secondary antibody (ab6721, 1:5000, Abcam, USA) was incubated at room temperature for 2 h.

### Citric acid and acetyl coenzyme A (acetyl-CoA) quantification

The femur and tibia were immersed in a mixture of chloroform and methanol (1:3 V/V) for approximately 1 h to degrease. Then, 2 mL of 1.0 M HCl was added to each 50 mg of ground bone to dissolve the hydroxyapatite [17]. Next, 100 µl of citrate detection buffer was added to the extract and centrifuged at 15000 × *g* for 10 min at 4 °C. The supernatant was removed and deproteinized with a perchloric acid/KOH protocol (Bio Vision, Cat. #K808-200). BMSCs were seeded into 6-well plates at a density of 1 × 10^6^ cells/well. After one week of culture according to the experimental design, 100 µl of lysate and buffer were added after the culture medium was aspirated. The contents of the wells were scraped into 1.5 ml EP tubes, centrifuged at 15000 × *g* for 10 min at 4 °C, and further deproteinized. The levels of citric acid in bone and cell samples were determined according to the procedures of the colorimetric/fluorescence assay kit (Bio Vision, Cat. #K655-100). In addition, acetyl-CoA in cells were detected according to the manufacturer’s instructions (BC0980; Solarbio).

### ALP and Alizarin red staining

Cells were fixed in 4% paraformaldehyde for 20 min and washed three times with PBS after the 7th day of culture. Next, the cells were stained with a 5-bromo-4-chloro-3-indolyl phosphate (BCIP)/nitro blue tetrazolium (NBT) ALP Detection kit (Beyotime, Shanghai, China). For ALP quantification, the original medium was removed, and the cells were washed three times with PBS and lysed at 4 °C for 30 min. After removing the whole cells and cellular debris via centrifugation at 2000 × *g* for 10 min at 4 °C, the supernatant was collected and distributed into each well of 96-well plates, and the absorbance was measured by a microplate absorbance reader (Bio‑Rad) at 520 nm. After 21 days of culture according to the experimental design, the medium was removed. The cells were washed three times with PBS, fixed with 95% ethanol for 15 min, and incubated with 0.1% alizarin red for 15 min. After removal of the staining solution, the cells were washed 5 times with PBS, and the mineralized nodules were observed by an inverted microscope. In addition, 1.0 M HCl was added to each well to dissolve the mineralized nodules, and citric acid was further detected following the methods described in Citric acid and acetyl coenzyme A (acetyl-CoA) quantification.

### Micro-CT

Microcomputed tomography (Micro CT, SkyScan1276, Bruker, Germany) was used to detect bone mineral density (BMD), bone volume/tissue volume (BV/TV), trabecular number (Tb.N), trabecular separation (Tb.sp), trabecular thickness (Tb.th) and structure model index (SMI) of the femur. The scanning parameters were a source voltage of 55 kV, a source current of 72 uA, a tomographic angle of 180° and a rotation step of 0.4°.

### Seahorse-based cell respiration analysis

A Seahorse XFp analyser (Agilent Seahorse Biosciences, North Billerica, MA, USA) was used to detect the oxygen consumption of BMSCs to reflect mitochondrial function. BMSCs were seeded in a Seahorse mini plate at a density of 3 × 10^4^ cells/well with osteogenic induction, H_2_O_2_ and MMP-9-IN-1 stimulation. Prior to analysis, the medium was replaced with Seahorse XF buffered base medium (Agilent, Santa Clara, CA, USA) supplemented with 2 mM glutamine, 1 mM pyruvate and 5.5 mM glucose at a pH of 7.4 and balanced in a CO_2_-free incubator at 37 °C for 1 hour, followed by sequential treatment with 1 μM oligomycin, 2 μM FCCP, and 0.5 μM rotenone/antimycin A. The results were normalized to the cell number in each well and analysed by Seahorse Wave Desktop Software.

### Statistics

The data are expressed as the mean ± standard deviation (SD) and were analysed using GraphPad Prism 8 (San Diego, CA, USA) and SPSS 22.0 (Chicago, IL, USA). ImageJ (NIH, MD, USA) was used to quantify protein bands. The Kolmogorov‒Smirnov test was used to verify the normality of the data. Student’s *t*-test or one-way ANOVAs and Tukey’s post hoc test were used to analyse differences between groups. *P* < 0.05 was considered statistically significant. All cell experiments were repeated three times.

### Supplementary information

Original Western Blots

## Data Availability

The datasets used and/or analysed during the current study are available from the corresponding author on reasonable request. R scripts for PCA analysis is available for download at https://CRAN.R-project.org/package=factoextra and https://CRAN.R-project.org/package=FactoMineR. R scripts for violin chart visualization is available for download at https://cran.r-project.org/web/packages/ggplot2/index.html. R scripts for orthogonal partial least squares discriminant analysis (OPLS-DA) is available for download at https://github.com/xia-lab/MetaboAnalystR. R scripts for Pearson correlation coefficient analysis is available for download at https://cran.r-project.org/web/packages/ggcorrplot/index.html. R scripts for enrichment analysis is available for download at https://github.com/YuLab-SMU/clusterProfiler.
