# Supplementary material for: ROS/MMP-9 mediated CS degradation in BMSC inhibits citric acid metabolism participating in the dual regulation of bone remodelling
Source: Cell Death Discov. 2024 Feb 14;10:77. doi: 10.1038/s41420-024-01835-5 (PMC10866869; doi:10.1038/s41420-024-01835-5)
Supplement: Supplementary file 1 — Original Western Blots [file 41420_2024_1835_MOESM1_ESM.docx]

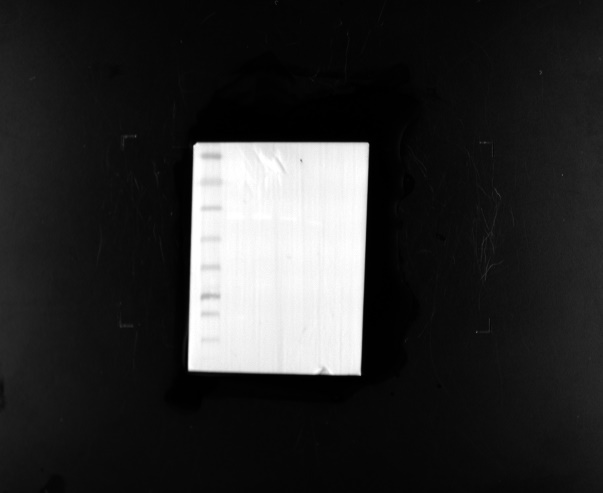


3 repeats of the original picture of the Western blotting of MMP-9 of Fig.4D.







3 repeats of the original picture of the Western blotting of MMP-9 of Fig.4D.




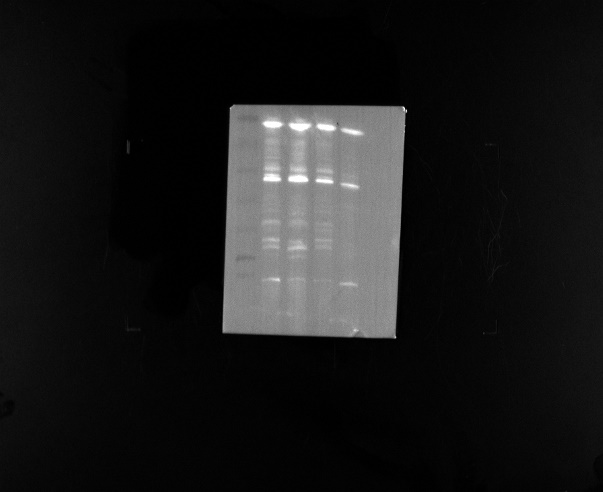


3 repeats of the original picture of the Western blotting of MMP-9 of Fig.4D.







3 repeats of the original picture of the Western blotting of CS of Fig.4D.







3 repeats of the original picture of the Western blotting of CS of Fig.4D.







3 repeats of the original picture of the Western blotting of CS of Fig.4D.




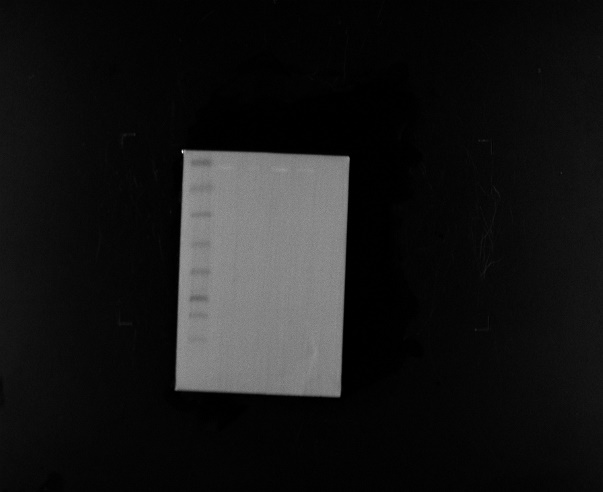


3 repeats of the original picture of the Western blotting of H3K9ac of Fig.4D.




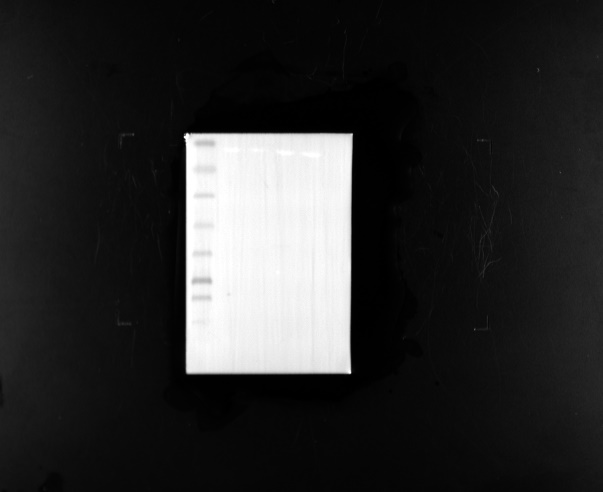


3 repeats of the original picture of the Western blotting of H3K9ac of Fig.4D.




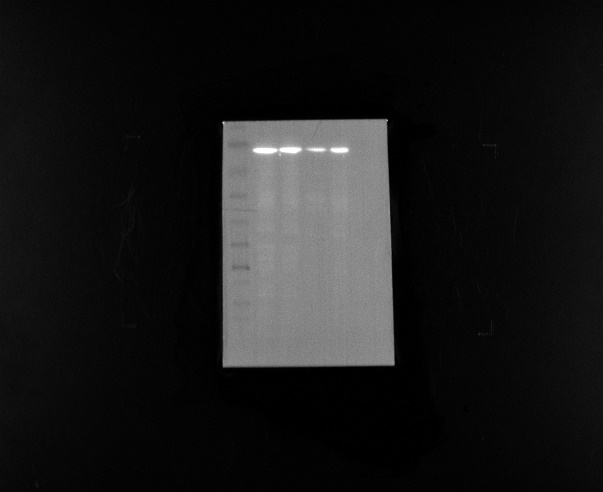


3 repeats of the original picture of the Western blotting of H3K9ac of Fig.4D.







3 repeats of the original picture of the Western blotting of H3 of Fig.4D.







3 repeats of the original picture of the Western blotting of H3 of Fig.4D.







3 repeats of the original picture of the Western blotting of H3 of Fig.4D.







3 repeats of the original picture of the Western blotting of Runx2 of Fig.4D.







3 repeats of the original picture of the Western blotting of Runx2 of Fig.4D.







3 repeats of the original picture of the Western blotting of Runx2 of Fig.4D.




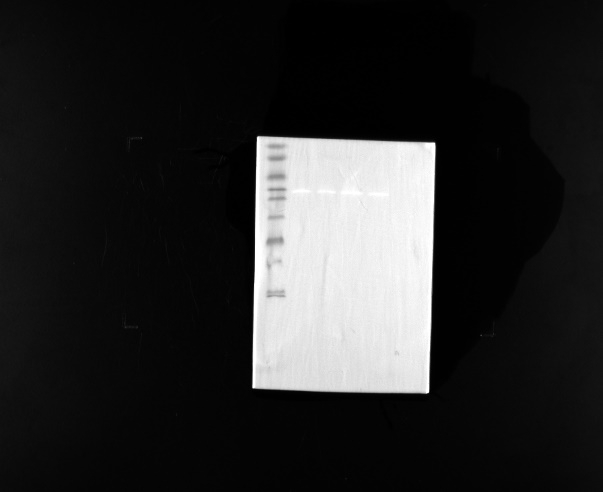


3 repeats of the original picture of the Western blotting of GAPDH of Fig.4D




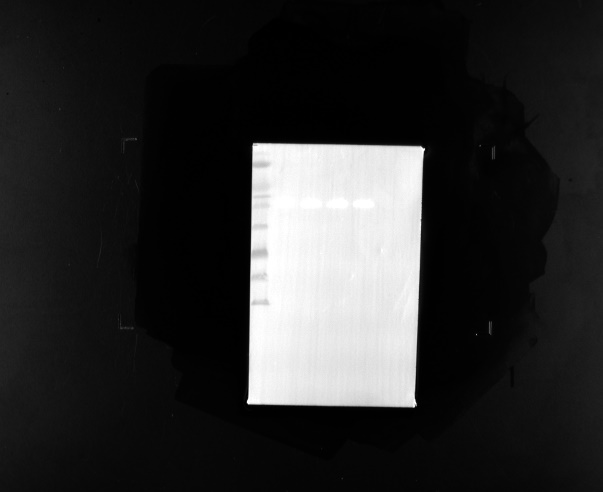


3 repeats of the original picture of the Western blotting of GAPDH of Fig.4D




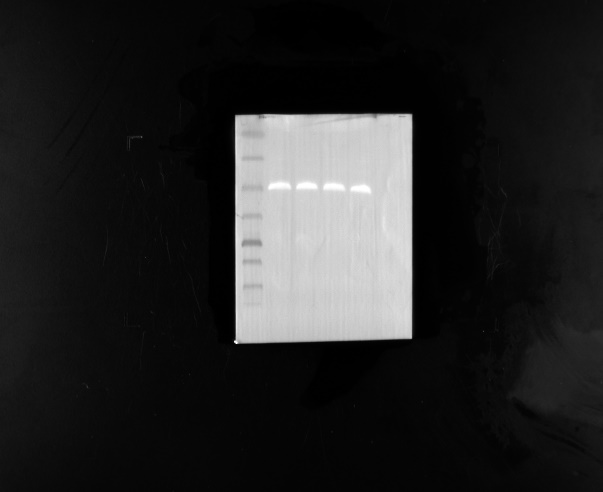


3 repeats of the original picture of the Western blotting of GAPDH of Fig.4D
